# Let’s Give Together: Can Collaborative Giving Boost Generosity?

**DOI:** 10.1177/08997640221074699

**Published:** 2022-02-12

**Authors:** Jason D. E. Proulx, Lara B. Aknin, Alixandra Barasch

**Affiliations:** 1 Simon Fraser University, Burnaby, British Columbia, Canada; 2 New York University, Stern School of Business, New York City, New York, USA

**Keywords:** generosity, charitable giving, collaboration, intrinsic motivation

## Abstract

A growing number of people donate to charity together with others, such as a spouse, friend, or stranger. Does giving to charity collectively with another person—called *collaborative giving*—promote generosity? Existing data offer unsatisfactory insight; most studies are correlational, present mixed findings, or examine other concepts. Yet, theory suggests that collaborative giving may increase generosity because giving with others could be intrinsically enjoyable. We conducted two well-powered, pre-registered experiments to test whether collaborative giving boosts generosity. In Experiment 1 (*N* = 202; 101 dyads) and Experiment 2 (*N* = 310; 155 dyads), pairs of unacquainted undergraduates earned money and were randomly assigned to donate collaboratively (Experiments 1–2), individually in each other’s presence (Experiments 1–2), or privately (Experiment 2). Across studies, we observed no condition differences on generosity. However, collaborative (vs. individual) giving predicted greater intrinsic enjoyment, which, in turn, predicted larger donations, suggesting a promising potential mechanism for future research and practice.

Humans are one of the most prosocial species on the planet, and prosociality—the tendency to engage in kind acts to benefit others (e.g., charitable giving)—is a hallmark of a well-functioning society (Gintis et al., 2003; Rand & Nowak, 2013). Despite this exceptional capacity for prosociality, fewer people across the globe are giving their money and time to help others, and overall, donors are donating less than previous generations (Charities Aid Foundation, 2017; DoGood Institute, 2018; Giving USA, 2018, 2019; Rooney et al., 2018). As fewer people choose to donate, charities and nonprofits face an uncertain future and a diminishing capacity to serve their communities (e.g., CanadaHelps, 2018). Meanwhile, potential donors may forgo meaningful opportunities to enhance their happiness, social connection, and health (e.g., Aknin et al., 2019, 2020; Curry et al., 2018; Layous et al., 2017; Whillans et al., 2016;). Given the importance of prosociality for enhancing the quality of life and welfare of humanity, social scientists have spent many decades examining what promotes human generosity: from donor characteristics (e.g., Thielmann et al., 2020), the nature of charitable appeals (e.g., Small & Loewenstein, 2003; Whillans et al., 2017), to social context, including the mere presence of others (e.g., Dovidio & Penner, 2001; Reyniers & Bhalla, 2013; Wiesenthal et al., 1983). But does making a prosocial decision *collaboratively*, together with someone else, impact generosity? We examined the impact of this common yet unstudied phenomenon—*collaborative giving*—on generosity in two pre-registered experiments.

Past research has focused almost exclusively on the generosity of individuals giving independently, but people often give to charity with others. Cross-sectional data from 2,500 heterosexual couples in the United States show that over half of respondents (53%) regularly donate to charity with their spouse (e.g., Andreoni et al., 2003; Yörük, 2010). Friends and peers give to charity together, too. For example, over 1,500 “giving circles” exist in the United States—wherein small groups of individuals acting independently or as part of a larger network or formal organization pool their money and decide together how it should be donated (Collective Giving Research Group, 2017; Eikenberry & Bearman, 2009). Peer-to-peer fundraising and online crowdfunding through platforms, such as *Facebook Causes, GoFundMe*, and *Donors Choose*, have also gained popularity (e.g., Castillo et al., 2014; Chapman et al., 2019; Saxton & Wang, 2014). Data indicate that more than 20% of Americans engage in collaborative giving (Giving USA, 2018; Smith, 2016), and in 2017, *GoFundMe* reported a 33% increase in the number of donors using their platform—over 40 million (CanadaHelps, 2018).

How does this increasingly common form of giving impact generosity? Past theorizing offers competing accounts. On the one hand, collaborative giving could reduce generosity because people give less when they believe others will donate generously (e.g., Fischer et al., 2011; Wiesenthal et al., 1983). Moreover, because people are attuned to the thoughts and behaviors of others (Cialdini & Goldstein, 2004), low initial donation suggestions may set norms for collectives to give less (e.g., Croson & Shang, 2011). On the other hand, several theoretical perspectives suggest that collaborative (vs. solitary) giving could bolster generosity. Social pressure and normative information can motivate generosity: people often act more generously when their behavior is observable to others (e.g., Bradley et al., 2018) and when higher initial donation suggestions set norms of generosity (e.g., Reyniers & Bhalla, 2013). Critically, collaborative giving may spark one of the strongest drivers of human behavior—intrinsic motivation (Ryan & Deci, 2000). Giving to charity can fulfill humans’ basic psychological needs to feel autonomous, effective, and socially connected (Harbaugh et al., 2007; Zaki & Mitchell, 2011). Past work suggests that people are more intrinsically motivated by tasks that they complete collaboratively than alone (e.g., Carr & Walton, 2014; Isaac et al., 1999), possibly because sharing experiences with others can make positive experiences more enjoyable (e.g., Boothby et al., 2014). Because feeling intrinsically motivated can increase generosity (Gorczyca & Hartman, 2017; Ki & Oh, 2018), collaborative giving may boost generosity by amplifying intrinsic enjoyment.

Despite rich theoretical rationale suggesting that collaborative giving boosts generosity, the data are unclear. Some nationally representative panel data show that married American couples who give collaboratively give a larger percentage of their income to charities than couples who donate individually, suggesting that collaborative giving could bolster generosity (Toppe et al., 2002; Yörük, 2010). Yet, other large data sets find the opposite pattern: Married couples who give collaboratively give less of their income than couples who donate individually (Andreoni et al., 2003). These mixed findings leave the link between collaborative giving and generosity among married couples uncertain.

Collaborative giving in other relationships—such as between friends or peers—may be associated with greater generosity (e.g., Castillo et al., 2014). For example, members of giving circles report giving more to charity than those who donate individually (Carboni & Eikenberry, 2018; Eikenberry & Bearman, 2009), and significantly more of their income to charity than demographically matched peers (Carboni & Eikenberry, 2018). Of course, these data are open to alternative explanations, such as inflated self-reports and statistical confounds (e.g., household income). Most importantly, these data fail to explain whether participation in giving circles *causes* greater generosity or whether generous individuals seek out giving circles.

Gauging the causal impact of collaborative giving on generosity matters because charities often expend valuable resources to facilitate collaborative giving. For example, Bearman and Franklin (2018) found that a substantial number of charities reported considerable labor costs and financial challenges associated with hosting giving circles. Other reports suggest that charities can surpass recommended fundraising costs to enable collaborative giving (McGlone, 2017). Given the costs that facilitating collaborative giving can impose on nonprofits, it is worth interrogating the efficacy of this strategy. Even null results can offer invaluable practical guidance to practitioners contemplating how to use limited resources (Matosin et al., 2014). Moreover, given that the potential benefits of collaborative giving are grounded in well-established effects (e.g., observability, intrinsic motivation), null results can inform theories of generosity, especially when using rigorous, well-powered designs.

To our knowledge, only three experiments examine the causal impact of collaborative giving on generosity. Baron and colleagues (1974) found that participants were more generous when they made an individual donation decision compared with a group donation decision in a within-subjects design. However, this study had several methodological concerns: The within-subjects study design brought the two giving contexts in clear contrast. Furthermore, this study was likely underpowered with only 28 groups, the researchers removed data points, and the analyses did not model group dependency—that is, they did not statistically account for the fact that participants worked within groups within condition.

In a well-powered experiment, Bischoff and Krauskopf (2015) randomly assigned more than 400 participants to make a donation decision either independently or as a “collective” (group sizes unreported). Donations did not differ across conditions. However, communication was prohibited in the collective giving condition, thereby precluding a key defining feature of collaboration (e.g., Einolf et al., 2018). Moreover, effects may have been misestimated because group dependency was not modeled.

In the most recent and strongest experiment to date testing how collaborative giving impacts generosity, Harrell (2021) paired strangers (*N* = 150; *N* = 75 dyads) on Amazon MTurk and tasked them to make a series of giving decisions across individual and collaborative decision-making contexts. Each participant of the pair received a 10-token endowment—worth a bonus payment of up to US$2—and decided whether to give any of their tokens to another participant in the study other than their partner. In the first decision, participants individually chose how many tokens to give (vs. keep for themselves) and were told that their partner was making the same decision for a different recipient. Afterward, across two different decision-making contexts presented in random order, participants decided how to distribute their and their study partner’s combined 20 tokens between themselves and a different pair of participants. In the control context, participants made this decision individually, without talking to their study partner. In the experimental context, pairs spent 4 min sharing their thoughts with one another about the donation using a text-based online chat system and then were randomly assigned to make the decision either jointly or individually after the discussion. Participants were more generous when they could communicate with their partner (vs. not communicate) and when they made the giving decision jointly (vs. individually) after discussing it with their partner.

Notably, Harrell (2021) utilized a reasonably well-powered sample (75 dyads) and modeled group dependency in the analyses, thereby offering the most precise evidence to date that giving collaboratively with a stranger may boost generosity. However, although using a mixed design may have offered more statistical power, as described above, the within-subjects design may also have made the contrast between individual and collaborative giving especially salient. This contrast may give rise to demand characteristics and make giving collaboratively with another person seem particularly novel in comparison, encouraging participants to expend more effort which may artificially inflate generosity in turn (e.g., Inzlicht et al., 2018). Moreover, the participants in Harrell’s experiment used tokens to help other participants and discussed the giving decision using a text-based chat system. Although tokens and vouchers are often used in economic games to study generosity (Engel, 2011), these substitutes are less ecologically valid than donations using real money. Similarly, while people often discuss and make donations together using text-based chat systems (e.g., *GoFundMe*; Saxton & Wang, 2014), the social cues that facilitate interpersonal coordination and joint decision-making are often lost in text-based communication (Morrison-Smith & Ruiz, 2020 ; Reader & Holmes, 2016). As such, enabling in-person, face-to-face communication may establish a considerably more ecologically valid context in which to examine the impact of collaborative giving on generosity.

Critically, the conclusions of each of these articles come from single experiments that were not pre-registered and thus should be treated with caution. Indeed, researchers are now being encouraged to use pre-registered studies—wherein researchers declare their sample size and/or data collection stopping rule, hypotheses, and analytic plan prior to data collection and analyses—or registered reports—in which both the design and analytic plans undergo formal peer review prior to data collection. These methodological tools have become best practice in many sciences because precommitting to key decisions before examining the data dissuades researchers from altering their methodological or analytical plans in ways that inflate the chances of a false positive (Nosek et al., 2018, 2021; Simmons et al., 2011). For example, after analyzing their data, researchers may collect or exclude additional participants, which overturns undesirable results, or they may explore their data in various ways and later claim that these analyses were planned. Researchers are not precluded from exploring their data beyond their pre-registered plan. However, preregistration and registered reports confer greater evidentiary value because researchers are required to delineate findings that were hypothesized in advance from those that are exploratory and require further investigation.

Overall, given the conflicting evidence and limitations of past work, the impact of collaborative giving on generosity remains uncertain, understudied, and worthy of further investigation. We overcome the limitations of past research and causally examine the impact of collaborative giving on generosity using two well-powered, pre-registered experiments. To do so, we randomly assigned some pairs of unacquainted undergraduate peers to discuss and decide on a real donation together, while other pairs were randomly assigned to theoretically and practically relevant control conditions. In Experiments 1 and 2, some pairs were assigned to make an independent giving decision in a peer’s presence; this condition isolated the unique impact of collaborative discussion (i.e., joint decision-making) from the effect of mere social presence on generosity. In Experiment 2, we also included a private giving condition, wherein participants made a personal donation decision in private. Given that people are often solicited for donations independently in front of others or while alone (e.g., in a personal phone call), this condition benchmarks the value of collaborative giving against typical fundraising strategies. In both studies, we utilized a between-subjects design to eliminate the contrast between collaborative and individual giving inherent in a within-subjects design. We powered our studies to detect the smallest effect likely of interest to practitioners and scholars (*d* = .20; Cohen, 1988) and modeled dependency in our data, thereby providing precise estimates. To create an engaging and ecologically valid context, we enabled participants to make actual donations using real money and to make collaborative donations in person, face-to-face. Finally, we examined several potential variables that might explain the relationship between collaborative giving and generosity, with particular focus on intrinsic enjoyment as a mediator.

## Experiment 1

In Experiment 1, we randomly assigned pairs of participants to discuss and make a donation decision collaboratively (*collaborative giving* condition) or make a donation independently in a peer’s presence (*independent giving* condition). Our preregistration, materials, and data and code for Experiment 1 are on the Open Science Framework (OSF): https://osf.io/g93xr/?view_only=d74d8c4aadc74e67b2b8e8ae81d2cfe5.

Given the mixed evidence in the literature and because this was our first empirical investigation of our research question, we pre-registered that participants in the *collaborative giving* condition would donate more or less generously than participants in the *independent giving* condition (i.e., a nondirectional hypothesis).

We also pre-registered exploratory mediation analyses to examine whether intrinsic enjoyment explains the relationship between collaborative giving and generosity. We included several exploratory mechanisms and outcomes in our preregistration. However, because we did not find consistent results for these measures across studies, we do not report them here; the measures and data for these exploratory mechanisms and outcomes can be found on the OSF.

### Participants and Power

Power analyses indicated that 160 participants (80 dyads) would yield 90% power to detect our smallest effect size of interest, *d* = .20 (Lakens, 2017) at α = .05. We pre-registered our intention to collect data from 25% more participants for a total sample of 200 participants (100 dyads) to account for missing data. However, we later noticed an error in our power analyses (i.e., after Experiment 2 was conducted), indicating that we powered our sample to detect an α = .01. Thus, we have approximately 99% power to detect *d* = .20 at α = .05 in each experiment.

Our final sample of 202 undergraduates (101 dyads) participated in exchange for course credit (see Table 1 for demographics). Participants privately indicated their relationship with the other participant (e.g., friend, acquaintance, recognize but don’t know, stranger) in a questionnaire, enabling us to determine that dyads were predominantly strangers (96.2%); findings are the same when excluding friends/couples, so all dyads were included in analyses.

**Table 1.** Summary of Participant Demographics.

	Experiment 1 (*N* = 202)		Experiment 2 (*N* = 310)	
Demographic	*M* (*SD*)	*Mdn* (range)	*M* (*SD*)	*Mdn* (range)
Age	19.18 (1.88)	19.00 (17–29)	19.31 (2.56)	19.00 (17–41)
Sex/Gender	*N*	%	*N*	%
Male	57	28.2%	81	26.1%
Female	144	71.3%	228	73.6%
Non-binary	0	0.0%	0	0.0%
Other	0	0.0%	1	0.3%
Prefer not to answer	1	0.5%	0	0.0%
Race/Ethnicity	*N*	%	*N*	%
First Nation/Native American	0	0.0%	2	0.6%
African American/Black	6	3.0%	5	1.6%
Hispanic	3	1.5%	3	1.0%
Caucasian/White	64	31.7%	101	32.6%
Asian	99	49.0%	124	40.0%
Middle Eastern	9	4.4%	18	5.8%
Multiracial	11	5.4%	20	6.5%
Other^a^	8	4.0%	36	11.6%
Prefer not to answer	2	1.0%	1	0.3%

a A number of participants who responded “Other” for their race/ethnicity indicated “South Asian,” “Indian,” or “Punjabi” in Experiment 2 (*n* = 27).

### Procedure

Each dyad member first completed a baseline questionnaire in private (see questionnaires on the OSF). During this time, a research assistant (RA) randomly assigned the pair to either the *collaborative giving* or the *independent giving* condition.

After the survey, pairs in each condition sat side-by-side in a small meeting room. The RA told participants that they would receive a financial endowment for evaluating a video advertisement for a local charity, thereby introducing the charity and disguising our research question. Participants listed their thoughts and feelings about the advertisement in an adapted thought listing task (Cacioppo et al., 1997).

Critically, participants in the *collaborative giving* condition watched and evaluated the advertisement *together* for a joint CA$10 financial endowment and then discussed and made the donation decision together. Meanwhile, participants in the *independent giving* condition watched and evaluated the advertisement *individually* for a CA$5 financial endowment and made a personal donation decision in each other’s presence. Past work suggests that spending CA$5 or less on charity or someone else can have meaningful emotional rewards compared with spending equivalent amounts on oneself (Aknin et al., 2020; Dunn et al., 2008). Thus, we believed that CA$5/participant would be a consequential sum for participants. Financial endowments consisted of CA$1 coins, and all participants signed a receipt to increase perceptions of psychological ownership. A visible camera recorded participants’ behavior to confirm that they abided by experimental instructions.

Written instructions informed participants that they could donate all, some, or none of their endowment to charity. Participants deposited their donation into a small donation box. To encourage participants in the *collaborative giving* condition to jointly make the decision (and to compare donations per capita), participants were told that they would split whatever money was not donated evenly among themselves.

After the donation, participants privately completed a post-donation questionnaire assessing their intrinsic enjoyment among various exploratory measures.

#### Measures

We measured generosity as the amount of money each participant donated. Each participant could donate between CA$0 and CA$5.

To ensure interactions were in line with condition assignment, both participants reported how much time they spoke with their peer when evaluating the advertisement and making their financial decision (0—*none of the time*; 4—*the whole time*).

Participants rated their intrinsic enjoyment during the donation decision on five items adapted from the Interest/Enjoyment subscale of the Intrinsic Motivation Inventory (i.e., IMI; “Our/My financial decision was fun to make”; “I thought making our/my financial decision was quite enjoyable”; α *=* .77; 1—*not at all true*; 7–*very true*; Ryan, 1982).

#### Results

A nested analysis of variance (NANOVA) confirmed the success of our manipulation: Participants in the *collaborative giving* condition reported spending more time talking with their study partner about the donation than participants in the *independent giving* condition (see Table 2).

**Table 2.** Summary of Analyses (Experiment 1).

Measure	Independent *N* = 100 *M* (*SD*)	Collaborative *N* = 102 *M* (*SD*)	*F*_Condition_	*p*	$\eta_{p}^{2}$ [90% CI]
Manipulation check	0.17 (.43)	2.02 (1.12)	*F*(1, 99) = 226.30	<.001	.70 [.61, .75]
Generosity (CA$)	4.42 (1.41)	4.66 (.95)	*F*(1, 99) = 1.50	.223	.02 [.00, .08]
Intrinsic enjoyment	4.59 (1.26)	5.14 (1.15)	*F*(1, 99) = 9.86	.002	.09 [.02, .19]

*Note.* CI = confidence interval.

**Figure 1.**
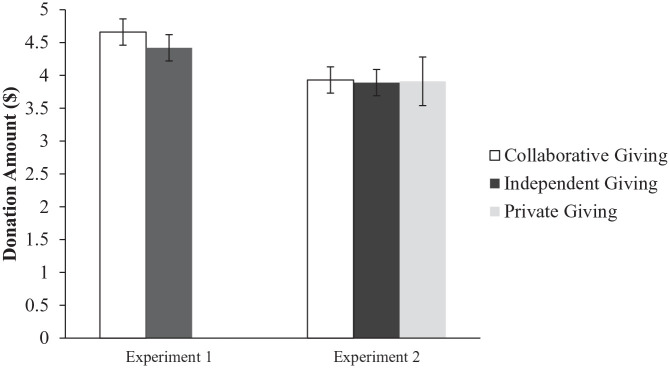
Average donation rates across conditions in Experiments 1 and 2. *Note.* Error bars represent 95%CIs. CI = confidence interval.

As pre-registered, we tested whether participants in the *collaborative giving* condition donated differently than participants in the *independent giving* condition. NANOVAs revealed no condition differences in generosity, *F*(1, 99) = 1.50, *p* = .223, $\eta_{p}^{2}=.02$ , 90% confidence interval (CI) [.00, .08] (see Table 2); the results remained unchanged when controlling for demographics or examining potential moderation effects by biological sex (see Supplemental Online Materials [SOM]). Participants in the *collaborative giving* condition donated directionally, but not significantly, more than participants in the *independent giving* condition (see Figure 1). This null effect may have been due to a ceiling effect: 85% of the sample donated the full CA$5 to charity.

Despite no significant direct effect, we followed the guidance of Hayes and Rockwood (2017) and Zhao and colleagues (2010) to examine whether there was an indirect effect of condition on generosity through intrinsic enjoyment. Indeed, because both (a) suppressor effects and/or competing direct and indirect effects can obscure total effects and (b) inferential statistics can be conducted on indirect effects themselves, indirect effect analyses provide meaningful insight into mediational processes when no significant total effect exists (Hayes, 2009; Hayes & Rockwood, 2017; Kenny & Judd, 2014). We first tested whether collaborative giving was more intrinsically motivating than giving independently in a peer’s presence. Participants in the *collaborative giving* condition reported greater intrinsic enjoyment than participants in the *independent giving* condition, *F*(1, 99) = 9.86, *p* = .002, $\eta_{p}^{2}=.09$ , 90% CI [.02, .19] (see Table 2).

**Figure 2.**
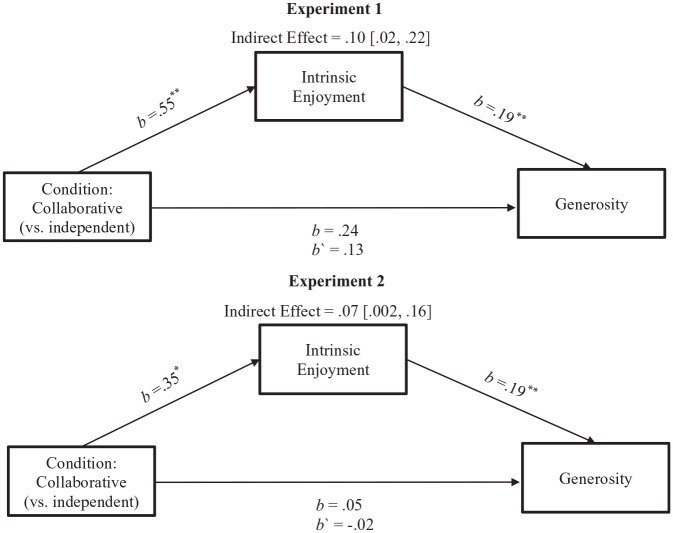
The indirect effect of collaborative giving (vs. independent giving) on generosity through intrinsic enjoyment in Experiments 1 and 2. *Note.* All *b*s represent unstandardized regression coefficients estimated using multilevel modeling (MLM) with maximum likelihood estimation. The indirect effect was obtained using *RMediation*. The range in brackets represents the 95% confidence interval of the indirect effect. *p < .05. **p < .01; otherwise, p > .05.

We then tested for an indirect effect of intrinsic enjoyment using multilevel linear modeling with participants at Level 1 clustered within dyads at Level 2 to estimate each path in the mediation model. Specifically, using maximum likelihood estimation, we estimated the effect of condition (Level 2) on intrinsic enjoyment (path a) and the effect of intrinsic enjoyment (grand mean centered) on generosity (path b). We tested the indirect effect using *RMediation* (Tofighi & MacKinnon, 2011). We found a significant indirect effect of intrinsic enjoyment, *b* = .10, 95% CI [.02, .22], suggesting that giving collaboratively with a peer (vs. independently in a peer’s presence) may boost generosity through greater intrinsic enjoyment (Figure 2).

#### Discussion

A pre-registered, well-powered experiment revealed that donating together with a peer did not significantly influence generosity beyond donating independently in a peer’s presence. This null effect may have resulted from exceptionally high donation rates across conditions. However, exploratory analyses revealed an interesting possibility: donating collaboratively with a peer (vs. independently in a peer’s presence) may cultivate greater intrinsic enjoyment which may then boost generosity.

We conducted a second well-powered, pre-registered experiment to investigate these findings more deeply. Specifically, in Experiment 2 we aimed to minimize the likelihood of a ceiling effect to better detect condition differences in generosity should they exist through several procedural changes. First, because observability can lead to greater generosity (e.g., Bradley et al., 2018; Kraft-Todd et al., 2015), we replaced the visible video camera with a covert camera in Experiment 2. Second, because our emotionally gripping charitable video advertisement may have prompted high levels of sympathy (e.g., Small, 2011; Small et al., 2007), we used a print ad in Experiment 2. Third, in Experiment 2, we notified participants of their payment upon registration, allowing participants to mentally budget the funds elsewhere (e.g., lunch) and feel a greater sense of psychological ownership, which should make the money more difficult to donate (e.g., Pierce et al., 2003).

Moreover, in Experiment 2, we added a *private giving* condition wherein each member of a pair was randomly assigned to make a personal donation in a private room. As people often donate or are solicited for donations in private, this condition provided a helpful benchmark for the potential benefits or costs of collaborative giving. Finally, Experiment 2 enabled us to conduct a confirmatory test of whether collaborative giving indirectly boosts generosity through greater intrinsic enjoyment.

## Experiment 2

In Experiment 2, pairs of participants were randomly assigned to discuss and make a donation decision collaboratively (*collaborative giving* condition), make a donation decision independently in a peer’s presence (*independent giving* condition), or make their own private donation decision (*private giving* condition). Our preregistration, materials, and data and code for Experiment 2 are on the OSF: https://osf.io/g93xr/?view_only=7005dcbc99f6478ea0fa68f27d3ea5cb.

In Experiment 1, collaborative giving had no effect on generosity relative to giving independently in a peer’s presence, but exploratory analyses suggested that collaborative giving may indirectly boost generosity by boosting intrinsic enjoyment. Thus, we pre-registered that participants in the *collaborative giving* (vs. *independent giving*) condition would donate more generously (H_1_), report greater intrinsic enjoyment from making the donation decision (H_2_), and that greater intrinsic enjoyment would mediate the relationship between condition and generosity (H_3_).

We made no a priori predictions about the levels of generosity or intrinsic enjoyment of participants in the *private giving* condition. We pre-registered nondirectional tests to compare participants in the *private giving* condition with participants in our two primary conditions.

### Methods

#### Participants and power

Power analyses indicated that a new sample of 240 participants (120 dyads) would yield 90% power to detect *d* = .20, with α = .05, one-tailed. We pre-registered a target sample of 300 participants (150 dyads) to account for up to a 25% attrition rate. As previously mentioned, we noticed after Experiment 2 was conducted that we mistakenly powered our sample to an α = .01; thus, we have approximately 99% power to detect our effect size of interest (*d* = .20) at α = .05. Because several participants were scheduled in advance of reaching our target sample size, we recruited a final sample of 310 undergraduates (155 dyads) using identical recruitment procedures as Experiment 1 (see Table 1 for demographics). As in Experiment 1, removing friends or acquaintances (8.1%) did not substantively change the results, and so they were left in the analyses.

### Procedure

Experiment 2 was largely identical to Experiment 1 with a few key changes. Participants received and signed for their CA$5 payment—which they knew about upon study registration—before placing it in their wallets, pockets, or bags. Payments were paid in smaller denominations (i.e., 1 × CA$2 coin, 2 × CA$1 coins, 4 × CA$0.25 coins) to enable greater variability in donations (e.g., CA$2.50, CA$1.75) than Experiment 1. After privately completing a baseline questionnaire, pairs of participants listed their thoughts and feelings about a print advertisement for the same local charity as Experiment 1; pairs randomly assigned to the *collaborative giving* condition did this task together, whereas pairs in the *independent giving* and *private giving* conditions did so individually. Unlike Experiment 1 where participants read the manipulation instructions, an experimenter kept blind to study hypotheses told pairs in the *collaborative giving* condition to (a) pool their payments and (b) discuss and decide together how much of their combined CA$10 to donate to the charity (vs. equally split between themselves). Meanwhile, participants in the *independent* and *private giving* conditions decided individually how much of their CA$5 to donate (vs. keep). We confirmed that participants in the *collaborative giving* and *independent giving* conditions complied with study instructions through covert video recording; we prioritized recording our primary two conditions because limited funds prevented us from purchasing cameras for each testing room. Participants deposited their donation in a donation box and then privately completed a post-donation questionnaire assessing their intrinsic enjoyment and several exploratory measures.

### Measures

We measured generosity as the amount of money each participant donated. Approximately 10% of the sample (*N* = 32) gave their own money in addition to the money from the study payment, meaning that our measure of generosity ranged between CA$0 and CA$10 rather than CA$5.

We measured intrinsic enjoyment using the same items adapted from the IMI (α = .79; Ryan, 1982) as in Experiment 1, but we removed one item to shorten the scale and reduce participant burden. We also made some minor language changes for clarity and consistency (i.e., “donation” vs. “financial” decision; “the other participant” vs. “study partner”).

### Results

A NANOVA omnibus test revealed a significant effect of condition on the time pairs spent discussing the donation decision, *F*(2, 151) =193.16, *p* < .001, $\eta_{p}^{2}=.72$ , 90%CI [.66, .76] (see Table 3). As recommended, we used Fisher’s least significant difference (LSD) to control family-wise error in planned pairwise comparisons (Myers et al., 2010). Confirming the success of the manipulation, participants in the *collaborative giving* condition discussed their donation more than participants in either the *independent giving* or *private giving* condition (*p*s < .001; see Table 3). On average, pairs in the *independent* and *private giving* conditions spoke very little, but participants in the *private giving* condition reported speaking significantly less (*p* < .001). This suggests that although there was minimal conversation between peers who completed the task separately in the same room, conversation was effectively zero among peers placed in individual testing rooms.

**Table 3.** Summary of Analyses (Experiment 2).

Measure	Collaborative (*N* = 102) *M* (*SD*)	Independent (*N* = 104) *M* (*SD*)	Private (*N* = 104) *M* (*SD*)	Omnibus *F*_Condition_	*p*	$\eta_{p}^{2}$ [90% CI]
Manipulation check	2.65 (1.25)	.64 (.88)	.05 (.26)	*F*(2, 151) = 193.17	<.001	.72 [.66, .76]
Generosity (CA$)	**3.93**^a,b^ **(1.59)**	**3.89**^a,c^ **(1.93)**	3.90^b,c^ (1.92)	*F*(2, 152) = .01	.988	.00 [.00, .00]
Intrinsic enjoyment	**4.77**^d^ **(1.30)**	**4.42**^e^ **(1.38)**	4.40^d,e^ (1.42)	*F*(2, 151) = 2.51	.085	.03 [.00, .08]

*Notes*. ^a–e^ Means that share a common superscript do not differ (*p* > .05). Pairwise comparisons between bolded means were pre-registered to be directional (one-tailed); all other comparisons are nondirectional (two-tailed). CI = confidence interval.

As pre-registered, we used a NANOVA and planned, one-tailed LSD pairwise comparisons to examine whether participants in the *collaborative giving* condition were more generous than participants in the *independent giving* condition. Analyses revealed no condition differences in generosity, *F*(2, 152) = 0.12, *p* = .988, $\eta_{p}^{2}=.00$ , 90% CI [.00, .01] (see Table 2). As in Experiment 1, participants in the *collaborative giving* condition gave directionally, but not significantly, more than participants in the *independent giving* condition (*M*_diff._ = 0.04, *SE* = 0.21, *p* = .420; see Figure 1) and the results remained unchanged when controlling for demographics or examining potential moderation effects by biological sex. Thus, these results fail to support the hypothesis that collaborative giving among unacquainted peers directly boosts generosity. This null result is informative because, unlike Experiment 1, we did not observe a ceiling effect on donations.

We next tested whether giving collaboratively with a peer was more enjoyable than giving independently in the presence of a peer. The omnibus test did not reach significance, *F*(2, 151) = 2.51, *p* = .085, $\eta_{p}^{2}=.03$ , 90% CI [.00, .08]. Despite this result, we tested our pre-registered directional hypothesis that participants in the *collaborative giving* condition would report greater levels of intrinsic enjoyment than participants in the *independent giving* condition. In line with this hypothesis—and replicating results from Experiment 1—participants in the *collaborative giving* condition reported greater levels of intrinsic enjoyment than participants in the *independent giving* condition (*M*_diff._ = 0.35, *SE* = 0.29, *p* = .035; see Table 3).

Despite the null result of condition on generosity, we tested our pre-registered prediction that participants in the *collaborative giving* (vs. *independent giving*) condition would be more generous through greater intrinsic enjoyment (Hayes & Rockwood, 2017; Zhao et al., 2010). We used the same analytic strategy as Experiment 1, using one-tailed tests to align with our directional hypotheses; *independent giving* = 0; *collaborative giving* = 1. In line with our pre-registered hypothesis—and replicating Experiment 1—we found a significant indirect effect of intrinsic enjoyment, *b* = .07, 90% CI [.002, .16] (see Figure 2). These findings suggest that, compared with donating individually in the presence of a peer, donating collaboratively with a peer may indirectly boost generosity through greater intrinsic enjoyment.

We found no significant differences in the level of generosity displayed between participants in the *private giving* condition and the two other conditions (*p*s > .864; two-tailed). The lack of difference between the *private giving* and *collaborative giving* conditions suggests that asking people to give collaboratively with or in the presence of a peer has no impact on donations beyond more typical, private giving practices. Interestingly, the lack of differences in generosity between participants in the *private giving* and *independent giving* conditions suggests that being observed by another participant in the *independent giving* condition did not raise donations in the present study either.

Nondirectional pairwise comparisons revealed that participants in the *collaborative giving* condition reported marginally more intrinsic enjoyment than participants in the *private giving* condition (*p* = .058); participants reported similar levels in the *private giving* and *independent giving* conditions (*p* = .923). Thus, giving collaboratively *with* a peer may be marginally more intrinsically enjoyable than giving in private, but giving privately may be as intrinsically enjoyable as giving individually in a peer’s presence.

Finally, we tested for an indirect effect of intrinsic enjoyment comparing participants collapsed across the *private* and *independent giving* conditions with participants in the *collaborative giving* condition (0 = *private giving* and *independent giving*; 1 = *collaborative giving*). We initially pre-registered the following model: –1 = *private giving*; 0 = *independent giving*; 1 = *collaborative giving*. However, because participants in the *private giving* and *independent giving* conditions did not differ on intrinsic enjoyment or generosity, collapsing across the *private giving* and *independent giving* conditions offers a stronger, more interpretable test. Consistent with the overall hypothesis, analyses revealed a significant indirect effect of intrinsic enjoyment, *b* = .10, 95% CI [.01, .22], 90% CI [.02, .20].^1^ Thus, these results suggest that giving collaboratively with a peer may indirectly boost generosity through greater intrinsic enjoyment compared with giving individually.

In the SOM, we report several additional exploratory analyses across experiments. Specifically, we probed whether social pressure potentially suppressed a direct effect of condition on generosity. We additionally coded the videos of participants in the collaborative giving condition and examined how various dimensions of the content and style of communication between participants predicted generosity and intrinsic enjoyment. Finally, we probed whether interpersonal closeness between peers may drive greater intrinsic enjoyment reports in the *collaborative giving* condition.

Overall, we found no consistent pattern across studies suggesting that social pressure suppressed a direct effect of condition on generosity. We similarly found no evidence that the style and content of communication between peers making a collaborative giving decision reliably predicted changes in generosity. However, peers coded as having a higher quality interaction (e.g., a deeper and livelier back-and-forth conversation) reported feeling greater intrinsic enjoyment. Moreover, we found preliminary evidence that feelings of interpersonal connection may mediate the relationship between condition and intrinsic enjoyment, suggesting one potential reason why giving collaboratively with a peer may be more intrinsically rewarding than individual forms of giving: It may help peers form meaningful social bonds with one another. Importantly, these exploratory analyses do not alter the main interpretation of our main findings. However, they do offer intriguing avenues for future confirmatory work examining how social pressure, communication, and interpersonal closeness influence the relationships between collaborative giving, generosity, and intrinsic enjoyment.

## General Discussion

Does giving to charity collaboratively with a peer boost generosity? Across two well-powered, pre-registered experiments, we observed no difference in donations when people gave to charity with a peer compared with giving independently in the presence of a peer (Experiments 1 and 2) or privately (Experiment 2). However, across studies—including a pre-registered, confirmatory analysis—we found that people derive greater intrinsic enjoyment from making a collaborative donation with a peer, which may indirectly boost generosity.

The present work adds to the literature on the situational forces that shape human generosity (e.g., Dovidio & Penner, 2001; Schroeder et al., 1995) and offers novel insight into a relatively common, yet understudied form of charitable donations: collaborative giving. Past work has primarily focused on how a range of social factors—from mere presence to social norms—can affect generosity (e.g., Croson & Shang, 2011; Wiesenthal et al., 1983). This work extends this investigation to examine the potential consequences of two individuals joining together to donate as a cohesive unit, adding a new dimension to the contextual factors that may (or may not) influence giving.

Critically, our experiments robustly examine a meaningful instantiation of collaborative giving. Across experiments, two unacquainted peers used a collective CA$10 windfall and engaged in a brief conversation to make a joint donation. As noted above, we selected CA$10 as the monetary amount because past research has indicated that donations made with CA$5 or less can shift momentary affect (Aknin et al., 2020; Dunn et al., 2008). Therefore, paying CA$5 per person offers a feasible and consequential amount through which to experimentally examine collaborative giving. Similarly, recent data suggest that spending around 10 min in conversation with a stranger can be meaningful and perceived as “just right” (Sandstrom & Whillans, 2020). As such, the relatively brief conversations studied here offers a useful context for consideration. Notably, we recruited unacquainted pairs rather than pre-existing friends, romantic partners, or other strong ties because strangers and acquaintances can have important influences on our emotions and behavior (e.g., Epley & Schroeder, 2014; Flynn & Lake, 2008). Moreover, charities often invite people to give collaboratively with unknown others, such as a stranger or friend of a friend, in crowdfunding or peer-to-peer giving campaigns (e.g., Chapman et al., 2019). Thus, our work utilizes informed, tightly controlled, and well-powered methods to examine collaborative giving in a theoretically and practically important population.

Our findings conflict with prior work suggesting that collaborative giving increases short-term generosity (e.g., Collective Giving Research Group, 2017; Yörük, 2010). Because the methodological and analytical limitations of past experiments may have obscured the causal impact of collaborative giving (Baron et al., 1974; Bischoff & Krauskopf, 2015)—and more recent work was not pre-registered (Harrell, 2021)—the present research, which uses both rigorous methods and open science practices, provides valuable guidance for future investigation (cf. Matosin et al., 2014). Given past evidence and theory suggesting that collaborative giving may boost generosity, why did we not observe an effect?

One possibility for this null finding is that the effect of collaborative giving is so small that it would take a much larger sample to detect. That being said, we powered our experiments to detect the smallest effect size that we anticipated to be worthwhile for charities and scholars (*d* = .20; Cohen, 1988). Future work could employ Bayesian analyses to quantify the evidence for or against a null effect (Rouder et al., 2009; Wagenmakers, 2007).

It is also possible that short-term generosity is affected more strongly when people give collaboratively in a group or with a close other or “strong tie” (e.g., romantic partner, friend, or family member) rather than an unacquainted peer. While strangers often meaningfully influence people’s behaviors and emotions (e.g., Flynn & Lake, 2008; Sandstrom & Dunn, 2014), strong ties typically exert a more powerful influence on people’s behaviors (Felmlee & Sprecher, 2000; Kelley, 1983), including prosocial action (Bond et al., 2012). Various forms of collaborative giving involve close others, such as spouses or friends in giving circles (e.g., Eikenberry & Bearman, 2009; Yörük, 2010). Moreover, people in close relationships or part of larger groups are more likely to engage in longer, more in-depth conversations about giving than what might occur among strangers, which can subsequently influence generosity (e.g., Balliet, 2010; Kerr & Kaufman-Gilliland, 1994). Taken together, the type of relationship and number of people making a collective giving decision could explain why collaborative giving had no direct effect on generosity among unacquainted peers. Our work suggests that there may be interesting boundary conditions for when collaborative giving may or may not impact generosity and that researchers should consider additional theoretical factors, such as with whom people give. Future research should expand by studying close dyads, including romantic partners and friends, groups, and other forms of collaborative giving, such as giving circles or through online peer-to-peer platforms, to assess its potential in other forms.

Beyond the possibility of deeper conversations with close others, another reason that people may be more generous when giving with a strong tie is that they are motivated to maintain a positive image in the eyes of others. This concern may even alter how one behaves in front of acquaintances or strangers in the same social network, where reputation information could impact how one is treated in the future. If so, participants in the present studies may have inflated their generosity when visible or known to the other participant if they expected to see them again on campus (e.g., Bekkers, 2004; Hoffman et al., 1996). Although this possibility may be partially responsible for the ceiling effect observed in Experiment 1, this same explanation does not account for why individuals in the *Collaborative Giving* condition—where self-presentation concerns were likely most salient—did not offer larger donations to charity in both experiments.

A third possibility is that giving collaboratively with a peer may not affect how *much* people give in one-time donations, but how *often* people give to charity over time. Indeed, our focus on short-term generosity limits insight into people’s long-term prosocial behaviors and willingness to give in sustained ways over time. Research in Self-Determination Theory (Ryan & Deci, 2000) suggests that people are much more likely to engage in, sustain, and repeat behaviors that they find intrinsically enjoyable. Thus, if donating collaboratively is more intrinsically enjoyable, people may be motivated to continue giving collaboratively in the future (cf. Bruhin et al., 2020). Subsequent research can probe whether people may be more willing to engage in repeated acts of generosity—such as recurring donations or payroll deductions—when making decisions collaboratively with a peer rather than on their own.

Our findings suggest that collaborative giving may be a more intrinsically enjoyable form of giving, which aligns well with previous research. Indeed, working with others leads to greater intrinsic enjoyment and task engagement (e.g., Carr & Walton, 2014) and that greater intrinsic enjoyment leads to greater generosity (e.g., Bidee et al., 2013; Ki & Oh, 2018). These findings offer a potential explanation for why our results diverge from Harrell’s (2021) experiment, wherein participants gave more tokens to other participants when they communicated with and jointly made the giving decision with a study partner compared with when they made the giving decision independently. As previously mentioned, the mixed-subjects design may have made the contrast between individual and collaborative giving particularly salient. Because making a joint donation may have been especially novel and intrinsically enjoyable in comparison, this may have heightened the participants’ intrinsic enjoyment, allowing a direct effect of collaborative giving on generosity to surface. Of course, this possibility requires further investigation because mediational analyses cannot provide causal evidence of the overall relationship (e.g., Lemmer & Gollwitzer, 2017)— especially in the absence of a direct effect (Bullock et al., 2010). Thus, future work should use experimental causal chain designs to test causality directly (Spencer et al., 2005).

Along similar lines, our data are consistent with a large body of work documenting a robust association between intrinsic enjoyment and generosity (Aknin et al., 2019; Curry et al., 2018), but we are not able to determine the direction of causality in our data. The reason for this limited inference is twofold. First, best statistical practice suggests that it is ill-advised to reverse the outcome and mediator to evaluate the plausibility of one model over the other as each model shares an identical implied covariance matrix and thus does not offer different indices of fit (Lemmer & Gollwitzer, 2017; Thoemmes, 2015). Second, due to the nature of our experimental designs, we collected self-reports of our proposed mediator (intrinsic enjoyment) after measuring the outcome of generosity. Thus, we encourage future researchers to collect novel data and pre-register to test the relationship between collaborative giving and intrinsic enjoyment through generosity.

### Practical Applications

Given the substantive labor and financial costs associated with facilitating collaborative forms of giving (e.g., Bearman & Franklin, 2018; McGlone, 2017), the current findings suggest that charities might not want to invest precious resources helping strangers give collaboratively to bolster short-term generosity. However, collaborative giving did not *reduce* our participants’ generosity, and it *did* lead to more intrinsic enjoyment than standard forms of giving. Thus, charities might consider cost-effective strategies that leverage collaborative giving to indirectly bolster generosity through intrinsic enjoyment or consider other ways that related strategies (e.g., giving circles) can boost long-term generosity.

### Conclusion

The present research provides the first pre-registered and well-powered tests of whether giving collaboratively with a peer increases generosity over giving in the presence of a peer or privately. We found no direct impact of collaborative giving on generosity. However, collaborative giving did cultivate greater feelings of intrinsic enjoyment, which predicted higher levels of short-term generosity, suggesting an intriguing direction for future research. While generosity may be on the decline across the globe, more people may be willing to share the act of giving (e.g., through online communities). Thus, it critical to understand if and how giving with others can reward and facilitate the human capacity for prosociality.

## Research Data

sj-docx-1-nvs-10.1177_08997640221074699 – for Let’s Give Together: Can Collaborative Giving Boost Generosity? Click here for additional data file. sj-docx-1-nvs-10.1177_08997640221074699 for Let’s Give Together: Can Collaborative Giving Boost Generosity? by Jason D. E. Proulx, Lara B. Aknin and Alixandra Barasch in Nonprofit and Voluntary Sector Quarterly This article is distributed under the terms of the Creative Commons Attribution 4.0 License ( https://creativecommons.org/licenses/by/4.0/) which permits any use, reproduction and distribution of the work without further permission provided the original work is attributed as specified on the SAGE and Open Access pages ( https://us.sagepub.com/en-us/nam/open-access-at-sage).

## Notes

1. The multilevel modeling (MLM) regressing condition on intrinsic enjoyment failed to converge. Because we found no evidence of nonindependence for this path via the intraclass correlation coefficient, *ICC* (1,1) = −.03, 95% confidence interval [−.19, .13], *p* = .715, we conducted a linear regression (Kenny et al., 2006); the results did not alter conclusions.
