# Supplementary material for: Let’s Give Together: Can Collaborative Giving Boost Generosity?
Source: Nonprofit Volunt Sect Q. 2022 Feb 12;52(1):50–74. doi: 10.1177/08997640221074699 (PMC9829957; doi:10.1177/08997640221074699)
Supplement: sj-docx-1-nvs-10.1177_08997640221074699 – for Let’s Give Together: Can Collaborative Giving Boost Generosity? [file sj-docx-1-nvs-10.1177_08997640221074699.docx]

**Supplemental Exploratory Analyses (Experiments 1 & 2)**

**Controlling for Demographics**

We conducted exploratory analyses in which we repeated each of our primary tests across Experiment 1 and Experiment 2 while controlling for our collected demographics. We first controlled for each age, sex, and ethnicity in separate analyses and then entered all demographics into a simultaneous model. Given that each of these post-hoc analyses were not pre-registered, we took a conservative approach and employed two-tailed significance tests. See the Open Science Framework (OSF) for the data scripts for all exploratory analyses reported in this Supplemental Material: <https://osf.io/g93xr/?view_only=35b3e3f7c0e74ecc9dc418bd436c0940>.

First, using a Nested Analysis of Covariance (NANCOVA), we examined the impact of condition on generosity while controlling for demographics. For both Experiments 1 and 2, we observed no condition differences on generosity when we controlled for age, sex, and ethnicity separately or all demographics simultaneously, *p*s > .216.

We similarly used NANCOVA analyses to examine the impact of condition on intrinsic enjoyment while controlling for demographics. Again, controlling for demographics did not substantively change the conclusion of the results in Experiment 1: participants in the *collaborative giving* condition reported significantly greater intrinsic enjoyment than participants in the *independent giving* condition while holding demographics constant, *p*s < .009. However, in Experiment 2, because we employed two-tailed tests rather than a one-tailed test as in the manuscript, the interpretation of the results is shifted. Specifically, under a two-tailed test, giving collaboratively with a peer was only marginally more intrinsically motivating than giving independently in a peer’s presence or privately when controlling for demographics, *p*s < .071.

We examined the indirect effect of collaborative giving on generosity through intrinsic enjoyment while controlling for demographics. Consistent with the analytic strategy reported in the manuscript, we employed Multilevel Modelling (MLM) with maximum likelihood estimation to estimate each path but additionally entered all demographics simultaneously as fixed predictors in each model; 0 = *independent giving*; 1 = *collaborative giving*. We then entered the MLM-derived path estimates and their standard errors into *R*Mediation to calculate the indirect effects and their 95% confidence intervals. In Experiment 1, controlling for demographics did not change the conclusion of the results: participants in the *collaborative giving* (vs *independent giving*) condition gave indirectly more generously through greater intrinsic enjoyment, *b* = .10, 95%CI [.02, .21]. In Experiment 2, we tested our primary comparison—the *collaborative giving* and *independent giving* conditions—and found that condition was marginally associated with greater intrinsic enjoyment in the *collaborative giving* condition (*p* = .060) while controlling for demographics. Thus, we did not find a significant indirect effect of collaborative giving on generosity through intrinsic enjoyment when we controlled for demographics, *b* = .07, 95%CI [-.00, .18].

Finally, in Experiment 2, we additionally tested whether there was an indirect effect of intrinsic enjoyment after controlling for demographics when we compared participants collapsed across the *private giving* and *independent giving* conditions with participants in the *collaborative giving* condition (0 = *private giving* and *independent giving*; 1 = *collaborative giving*). However, our MLM analyses failed to converge when estimating the effect of condition on intrinsic enjoyment. We thus followed recommendations by Kenny and colleagues (2006) in conducting regression analyses with potential non-independence in the outcome variable. Specifically, we first examined whether there was significant non-independence in reports of intrinsic enjoyment using the recommended alpha level for assessing non-independence (*α* < .20). However, we found no evidence of significant non-independence in intrinsic enjoyment, *ICC*(1,1) = -.03, 95%CI [-.19, .13], *F*(153,154) = 1.06, *p* = .715. Thus, as recommended, we conducted a standard linear regression to estimate the effect of condition on intrinsic enjoyment while controlling for participant demographics. Given that our MLM analyses reached convergence for path b—that is, regressing generosity on both condition, intrinsic enjoyment (centered), and demographics—we utilized this MLM-derived estimate to calculate our indirect effect because MLM analyses offer greater statistical power. Analyses revealed a significant indirect effect of intrinsic enjoyment, *b* = .10, 95%CI [.01, .22].

Taken together, our exploratory analyses controlling for age, sex, and ethnicity did not substantively change the conclusion of our results. Across experiments, we observed no direct effect of condition on generosity when controlling for demographics separately or simultaneously. We did see condition differences in intrinsic enjoyment in Experiment 1, but these differences were marginal in Experiment 2 due to using two-tailed tests rather than one-tailed. Similarly, we observed the effect of collaborative giving indirectly boosting generosity through greater intrinsic enjoyment in Experiment 1 and again in Experiment 2 when comparing participants who gave collaboratively with those who gave individually—either privately or in the presence of a peer. Overall, these analyses suggest that when holding age, sex, and/or ethnicity constant, giving collaboratively with a peer may indirectly boost generosity through greater intrinsic enjoyment compared to giving individually.

**Sex/Gender Effects on Generosity**

Across our experiments, two undergraduates registered for each timeslot through the university subjects’ pool on a first-come, first-serve basis. As a result, participants formed either same sex dyads (i.e., female/female, male/male) or cross-sex dyads (female/male). Past work suggests that biological sex may meaningfully influence generosity in social situations. One prominent evolutionary theory known as *Competitive Altruism Theory* suggests that people may be motivated act generously in public at personal cost and compete with others to signal their worth and secure potential mates (Roberts, 1998). For example, Raihani and Smith (2015) demonstrated that heterosexual male donors compete with other males who give large public donations by giving even larger donations in response to a more (vs. less) attractive female fundraiser. However, female donors did not appear to compete with other female donors in response to an attractive male fundraiser (see also Sisco & Weber, 2019). Thus, it is possible that giving collaboratively with a peer may impact generosity differently depending on whether dyads are same- or cross-sex. For instance, to the extent that dyads are heterosexual, donations may be higher in cross-sex as compared to same-sex dyads—males may suggest a higher donation to look good in front of an attractive female partner. Alternatively, given that our experimenters were predominantly female, male-male dyads may donate more generously as compared to either female-female or cross-sex dyads because they may compete with one another to gain the favour of a female researcher.

While we did not assess the sexual orientation of our participants, we explored the intriguing idea that the composition of biological sex between dyads may moderate the effect of condition on generosity.^[[1]](#footnote-1)^ To maximize statistical power and aid in the interpretation of the analyses, we collapsed across same-sex dyads (i.e., female/female, male/male) and tested whether dyad type (0 = *same-sex dyads*; 1 = *cross-sex dyads*) interacted with condition to predict generosity across experiments; in Experiment 2, we additionally collapsed across the *independent giving* and *private giving* conditions (0 = *independent giving* and *private giving*; 1 = *collaborative giving*). In Experiment 1, we recruited 67% same-sex dyads (*n* = 56 female/female; *n* = 14 male/male) and 33% cross-sex dyads (*n* = 31) and in Experiment 2, we recruited 59% same-sex dyads (*n* = 83 female/female; *n* = 9 male/male) and 41% cross-sex dyads (*n* = 63); see Table S1 for the descriptive statistics on the generosity levels of each dyad-type. To test for moderation, we conducted MLM analyses regressing generosity on condition, dyad-type, and a dyad-type $\times$ condition interaction term. Across experiments, we found no evidence that the composition of biological sex between dyad members significantly moderated the effect of condition on generosity, *p*s > .161. Interestingly, in Experiment 1, accounting for the composition of biological sex and the interaction term left a marginally significant direct effect of condition on generosity such that giving collaboratively marginally predicted greater generosity, *b* = .42, *SE* = .23, *t*(101) = 1.83, *p* = .071. However, in Experiment 2, the direct effect of condition on generosity remained non-significant when modelling the moderation effect of the composition of biological sex between dyads, *p* = .688.

Overall, it seems unlikely that competitive altruism processes are operating such that giving collaboratively with a same-sex peer differentially impacts generosity than giving with a cross-sex peer. However, we were statistically underpowered to perform meaningful moderation analyses, so these results should be taken with caution and future researchers should continue to examine whether there is meaningful variation in how much people donate when they give collaboratively with a same- or cross-sex individual. That said, various elements of our design in Experiment 2 challenge the possibility that peers are engaging in costly signaling. Indeed, participants in the *private giving* condition who did not see or communicate with one another donated no differently than participants in either the *independent giving* or *collaborative giving* conditions, who could see their partner’s donation and/or communicate about the donation. Thus, because observability in general did not strongly shape participants’ donation behaviours, it is unlikely that people feel the need to signal greater generosity when they are donating in the presence of or together with a same- or cross-sex peer.

**Suppressor Effects**

Given the theoretical rationale and prior empirical work suggesting that collaborative giving can boost generosity, it is intriguing that we consistently observed that condition did not directly boost generosity but indirectly boost generosity through intrinsic enjoyment. Because unspecified moderation effects may have suppressed the direct effect (e.g., Hayes, 2009; Valeri & VanderWeele, 2013), we conducted post-hoc exploratory suppression effect analyses to examine this possibility.

One moderator that we assessed across experiments which may have suppressed a direct effect of condition on generosity might be social pressure. On the one hand, any social pressure felt by participants in the *collaborative giving* condition might *enhance* generosity (Bekkers & Wiepking, 2007). Indeed, a great deal of work suggests that people tend to act more generously when they are socially pressured to donate, such as when their giving behaviour is observable (e.g., Bradley et al., 2018). Moreover, because there is social value placed on generous action (Buss et al., 1990), people may be likely to communicate and follow a norm to act generously (e.g., Reyniers & Bhalla, 2013). On the other hand, feelings of social pressure may *diminish* generosity because social pressure may attenuate feelings of intrinsic motivation (e.g., Burgoyne et al., 2005). Indeed, past work suggest that when giving is not autonomous or socially pressured, people often do not experience the positive emotional rewards of giving (e.g., Harbaugh et al., 2007; Reyniers & Bhalla, 2013; Weinstein & Ryan, 2010) and the associated reduction in intrinsic motivation may attenuate both momentary and future acts of generosity (e.g., Reyniers & Bhalla, 2013; Ryan & Deci, 2000; Scott, 1976).

To test whether social pressure may suppress the effect of condition on generosity, we conducted MLM analyses in which we regressed generosity on both condition (0 = *independent giving*; 1 = *collaborative giving*), intrinsic enjoyment (centered), social pressure (centered), and a social pressure $\times$ condition interaction term. To maximize statistical power, we collapsed across the *independent giving* and *private giving* conditions in Experiment 2 (0 = *independent giving* and *private giving*; 1 = *collaborative giving*). Across experiments, analyses revealed no significant moderation by social pressure (*p*s > .303) and the direct effect of condition remained non-significant (*p*s > .505). In fact, participants who gave collaboratively consistently reported feeling *less* social pressure than participants who gave individually, though this difference did not reach significance in either experiment (*p*s >.153). Interestingly, in Experiment 1, greater social pressure predicted lower generosity, *b* = -.30, *SE* = .07, *t*(180.7) = -4.11, *p* < .001, and the effect of intrinsic enjoyment on generosity was attenuated, *b* = .09, *SE* = .07, *t*(194.1) = 1.24, *p* = .216. In Experiment 2, greater social pressure again predicted lower generosity, *b* = -.13, *SE* = .06, *t*(277.9) = -2.12, *p* = .035, however the effect of intrinsic enjoyment on generosity became substantially *larger*, *b* = .25, *SE* = .07, *t*(277) = 3.52, *p* = .001. Taken together, we observed no evidence that social pressure suppressed a direct effect of condition on generosity. While greater social pressure consistently predicted lower generosity across studies, social pressure did not moderate the effect of condition. Moreover, there was no consistent pattern across studies to suggest that social pressure had a competing effect on generosity with intrinsic enjoyment. Thus, additional work is needed to better understand what factors may suppress collaborative giving from having a direct effect on generosity and how social pressure may affect generosity when people give collaboratively with others.

**Communication and Generosity**

One of the defining features of collaborative giving, beyond joint decision-making, is the communication and negotiation that occurs between giving partners (Einolf et al., 2018). Past work suggests that communication can meaningfully influence prosocial behaviour (e.g., Balliet, 2010; Kerr & Kaufman-Gilliland, 1994), but little is known of whether the content or style of communication may affect generosity in collaborative giving contexts.

To better understand whether and how communication between peers may impact generosity, we had a team of four trained coders independently code the available videos of dyads within the *collaborative giving* condition across both Experiment 1 (*n* = 37 dyads)^[[2]](#footnote-2)^ and Experiment 2 (*n* = 45 dyads). Specifically, we had coders rate the conversations between pairs on several potentially revealing dimensions, including: (1) the amount of time pairs spent in conversation; (2) the proportion of time that pairs were engaged in task or decision relevant discussion; (3) the overall depth of conversation; and (4) the conversation dynamic between participants. Instances of collaborative giving often involve pairs discussing a charitable cause before they make a joint donation-decision, but sometimes people just discuss the joint allocation of money (e.g., Einolf et al., 2018). As such, coders separately rated each the advertisement evaluation and the joint-donation decision phases of the collaborative giving task on all four coding dimensions so that we could test the unique roles that each discussing the charitable cause and making the joint-decision have on generosity.

To assess the time pairs spent having conversation, a trained coder calculated the number of minutes that dyads were having conversation during each the advertisement evaluation and joint-donation decision phases. All coders additionally estimated the proportion of time that pairs spent having relevant and focused discussion during each phase of the experiment (1 = *0% Not at all focused*; 2 = *25%*; 3 = *50%*; 4 = *75%*; 5 = *100% Very focused*). Specifically, coders rated how much of the total time pairs spent discussing their thoughts or feelings about the advertisement or charity, their own or other people’s experiences with charity or children’s hospitals, their donation preferences, the consequences of their donation, and/or the reasons or emotions behind their decisions. To assess the overall depth of conversation, during each phase, coders rated to what extent participants were engaged in personal, genuine discussion—in which they shared detailed and/or sincere thoughts, emotions, or personal experiences with one another—or superficial, impersonal, brief discussion (1 = *Not at all deep*; 5 = *Very deep*). Finally, to capture the overall dynamic between peers, during each phase of the task, coders rated the extent to which pairs had lively, shared conversation—in which they asked each other questions and actively listened to and expanded on each other’s thoughts—or had a more one-sided conversation—in which the pair was not really engaged with one another, one person may have been driving the conversation, or peers shared mostly one-word responses like “Sure” or “Ok” (1 = *One-sided*; 5 = *Lively back-and-forth*). Across nearly every coding dimension, coders displayed acceptable to good reliability, *ICC*(2,4)s: .70 – .87; see Table S2. For the relevance dimension of the donation decision phase, reliability fell short of acceptable levels, *ICC*(2,4) = .69; we report the analyses for this dimension and encourage caution when interpreting the results.

As seen in Table S2, across experiments, pairs spent about five minutes discussing the advertisement and about a minute and a half making their joint donation decision. In each phase of the collaborative giving task, dyads across experiments had focused conversations, spending roughly 85 – 95% of the time having relevant conversation about the advertisement, their donation decision, the charity, their values, or personal experiences with charity. Overall, this conversation was rated at somewhat deep across experiments when dyads were evaluating the advertisement, with ratings at about the midpoint of the scale. However, when pairs were making their joint-donation decision, coders did not find that pairs had particularly in-depth conversations. Indeed, one-sample *t*-tests revealed that on average, coders ratings of conversation depth fell significantly below the midpoint of the scale during the joint-donation decision in both Experiment 1 (*M* = 1.97, *SD* = 1.00) *t*(35) = -6.17, *p* < .001, and Experiment 2 (*M* = 2.24, *SD* = .92), *t*(44) = -5.53, *p* < .001. Finally, across experiments, pairs seemed to have a somewhat lively conversation with one another, sometimes asking questions, actively listening to one another, and expanding on each other’s points. In general, pairs seemed to have moderately collaborative dynamic both when evaluating the advertisement—with coders rating the dyads about half a point above the scale midpoint across experiments. Similarly, coders rated the dynamic between pairs during the joint donation decision at the midpoint (Experiment 2) or just below (Experiment 1), suggesting that pairs seemed to be moderately engaged with one another as they made their mutual donation decision.

To examine whether the various dimensions of communication were associated with overall levels of generosity in dyads assigned to the *collaborative giving* condition, we conducted bivariate correlations between generosity (dyadic average) and each of our coding dimensions for both the advertisement evaluation and donation decision phases of the experiment (see Table S2 for correlations). Given the exploratory nature of these analyses, we employed two-tailed significance tests. As shown in Table S2, across experiments, the generosity of dyads in the collaborative giving condition was not associated with most of our coding dimensions in each phase of the experiment. Interestingly, in Experiment 2, spending more time having discussion when making the joint donation decision was moderately-to-strongly associated with less generosity, *r*(43) = -.48, *p* = .001. However, we observed no association between discussion time and generosity in Experiment 1, *r*(34) = -.09, *p* = .600.

To further probe the intriguing result that time spent talking with one’s peer may reduce generosity, we additionally tested whether our manipulation check was associated with participants’ generosity within the *collaborative giving* condition. Indeed, in each experiment, we captured participants’ self-reports of the proportion of time that pairs spent talking with each other across both the advertisement evaluation and joint donation decision phases. To test whether the proportion of time spent talking overall significantly predicted participants’ generosity within the *collaborative giving* condition, we conducted MLM analyses regressing participants’ generosity on their individual self-reports on the manipulation check. Interestingly, in Experiment 1, spending a greater proportion of time overall talking with one’s peer was associated with marginally less generosity, *b* = -.02, *SE* = .01, *t*(51.5) = -1.95, *p* = .057. However, unlike our analyses which suggested that the objective amount of time that pairs spent talking with one another during the joint-donation decision phase predicted less generosity in Experiment 2, self-reported time spent talking with one’s peer across both phases of the task did not predict generosity in Experiment 2, *b* = .09, *SE* = .10, *t*(73.3) = .91, *p* = .367.

Taken together, we observed no consistent patterns across experiments to suggest that the content and style of communication when discussing a charity or making a joint donation decision—particularly, how long peers spent in discussion, the overall depth and relevance of the conversation, or the conversational dynamic between peers—predicted changes in generosity. We observed an intriguing result that spending more time making the joint donation decision is associated with lower generosity. However, because we did not observe this result across studies, future confirmatory work is needed to probe this finding further and better understand why more conversation may predict lower generosity.

**Communication, Interpersonal Closeness, and Intrinsic Enjoyment**

Across experiments, we consistently observed that donating collaboratively with a peer was more intrinsically rewarding than donating individually—either independently in the presence of a peer or privately—and this boost in intrinsic enjoyment subsequently bolstered generosity. But why might sharing a donation decision with a peer be intrinsically rewarding? To examine this question, we repeated our exploratory correlation analyses above and examined whether the various dimensions of communication as rated by coders and the time pairs spent talking as self-reported by participants were associated with intrinsic enjoyment. To facilitate the interpretation of the analyses, for our dyadic-level predictors (i.e., coding dimensions) we conducted bivariate correlations with the dyadic average of intrinsic enjoyment. For our participant-level predictors (i.e., manipulation check), we conducted MLM analyses regressing individual participants’ intrinsic enjoyment scores onto their individual self-reports.

As seen in Table S2, in Experiment 1, the amount of time pairs spent in conversation during the advertisement evaluation phase as rated by coders predicted slightly greater intrinsic enjoyment, but this association was marginal, *r*(35) = .28, *p* = .099. However, there was no association between intrinsic enjoyment and the objective amount of time that pairs spent in conversation during the advertisement evaluation phase in Experiment 2, nor during the joint-donation decision phase across either experiment (*p*s > .131). Similarly, MLM analyses revealed that the self-reported proportion of time spent talking with one’s peer did not predict greater levels of intrinsic enjoyment across either experiment (*b*s < .13, *p*s > .191). Taken together, these analyses suggest that objective amount or subjective experience of time that participants spend talking with each other about a charitable advertisement or when making their joint decision does not lead to greater intrinsic enjoyment.

While the *quantity* of time that participants spend with one another may not lead to greater intrinsic enjoyment, it is possible that the overall *quality* of conversation can predict greater intrinsic enjoyment. Consistent with this possibility, across each experiment, conversation depth and the dynamic between pairs during the advertisement evaluation phase were moderately-to-strongly associated with greater intrinsic enjoyment (*r*s: .34 – .49). During the joint-donation decision phase in Experiment 1, depth of conversation marginally predicted greater intrinsic enjoyment, *r*(34) = .33, *p* = .051, and dynamic strongly predicted greater intrinsic enjoyment, *r*(34) = .52, *p* =.001. However, in Experiment 2, neither conversation depth nor dynamic during the joint donation-decision phase was significantly associated with intrinsic enjoyment (*p*s > .100). Taken together, these analyses reveal an intriguing possibility that when peers have a higher quality interaction marked by deeper conversation and a livelier back-and-forth dynamic—particularly when discussing a charitable cause—they experience greater intrinsic enjoyment from the collaborative giving experience.

Given that higher quality interactions may make an act of shared generosity more intrinsically rewarding, we reasoned that one possibility for why collaborative giving may be more intrinsically enjoyable than giving individually by a peer or in isolation is that it may create social bonds between peers. To test this possibility, we conducted exploratory multilevel mediation analyses to examine whether the effect of condition on intrinsic enjoyment is mediated through interpersonal closeness. We created generalized measures of interpersonal closeness of the five measures that were consistently used to assess interpersonal closeness in both Experiment 1 (α = .90) and Experiment 2 (α = .89). We accepted the high internal consistency as minimal justification to standardize and average across items to create overall composites of interpersonal closeness in each experiment.^[[3]](#footnote-3)^

We aimed to use MLM analyses to estimate the effect of each condition^[[4]](#footnote-4)^ on interpersonal closeness (path a) and interpersonal closeness (grand mean centered) on intrinsic enjoyment (path b) to calculate the indirect effect using *RMediation*. While the MLM models converged when estimating path a, in each experiment, the models failed to converge when estimating path b. As there was no evidence of non-independence in intrinsic enjoyment reports for either experiment (*ICC*s < .11; *p*s > .266), we followed recommended best practice (Kenny et al., 2006) and regressed individual participants’ reports of intrinsic enjoyment onto both condition and the interpersonal closeness composite (grand mean centered). As shown in Fig. S1, interpersonal closeness fully mediated the relationship between condition and intrinsic enjoyment in each Experiment 1, *b* = .39, 95% CI [.21, .61], and Experiment 2, *b* = .53, 95% CI [.33, .75], leaving non-significant direct effects (*ps* > .343). Specifically, compared to participants in the *independent giving* condition in Experiment 1 or across the *independent giving* and *private giving* conditions in Experiment 2, participants in the *collaborative giving* condition reported greater levels of interpersonal closeness which led to greater levels of intrinsic enjoyment.

Overall, our results suggest that one potential reason why giving collaboratively with a peer may be more intrinsically rewarding than giving independently around others or in solitary is because it offers a chance for peers to develop relationships with one another. Of course, it is also possible for the reverse model to be true: peers who engage in a fun, intrinsically enjoyable task together may develop greater social bonds as a result. Indeed, work suggests that engaging in novel, interesting, and enjoyable experiences with others can motivate people to expand themselves and approach and build connections with others (e.g., Aron et al., 2013; Fredrickson, 1998, 2001). However, we are unable to assess whether one model is more appropriate than the other with these data given that testing reverse mediational models is statistically inappropriate and often leads to inaccurate conclusions (Lemmer & Gollwitzer, 2017; Thoemmes, 2015). Rather, further confirmatory work with designs that manipulate both the focal factor and each mediator in the model is required (e.g., Spencer et al., 2005).

**References**

Aron, A., Lewandowski, G. W. Jr., Mashek, D., & Aron, E. N. (2013). The self-expansion model of motivation and cognition in close relationships. In J. Simpson & L. Campbell (Eds.), *The Oxford Handbook of Close Relationships* (pp. 90–115). Oxford University Press. https://doi.org/10.1093/oxfordhb/9780195398694.013.0005

Balliet, D. (2010). Communication and Cooperation in Social Dilemmas: A Meta-Analytic Review. *The Journal of Conflict Resolution*, *54*(1), 39–57. https://doi.org/10.1177/0022002709352443

Bekkers, R., & Wiepking, P. (2007). Generosity and philanthropy: A literature review. *SSRN Electronic Journal*. https://doi.org/10.2139/ssrn.1015507

Bradley, A., Lawrence, C., & Ferguson, E. (2018). Does observability affect prosociality? *Proceedings of the Royal Society B: Biological Sciences*, *285*(1875), 20180116. https://doi.org/10.1098/rspb.2018.0116

Burgoyne, C. B., Young, B., & Walker, C. M. (2005). Deciding to give to charity: A focus group study in the context of the household economy. *Journal of Community & Applied Social Psychology*, *15*(5), 383–405. https://doi.org/10.1002/casp.832

Buss, D. M., Abbott, M., Angleitner, A., Asherian, A., Biaggio, A., Blanco-Villasenor, A., Bruchon-Schweitzer, M., Ch’U, H.-Y., Czapinski, J., Deraad, B., Ekehammar, B., El Lohamy, N., Fioravanti, M., Georgas, J., Gjerde, P., Guttman, R., Hazan, F., Iwawaki, S., Janakiramaiah, N., … Yang, K.-S. (1990). International preferences in selecting mates: A study of 37 cultures. *Journal of Cross-Cultural Psychology*, *21*(1), 5–47. https://doi.org/10.1177/0022022190211001

Einolf, C. J., Curran, H. D., & Brown, K. C. (2018). How married couples make charitable giving decisions. *Nonprofit and Voluntary Sector Quarterly*, *47*(3), 657–669. https://doi.org/10.1177/0899764018757027

Fredrickson, B. L. (1998). What good are positive emotions? *Review of General Psychology*, *2*(3), 300–319. https://doi.org/10.1037/1089-2680.2.3.300

Fredrickson, B. L. (2001). The role of positive emotions in positive psychology: The broaden-and-build theory of emotions. *The American Psychologist*, *56*(3), 218–226. https://doi.org/10.1037//0003-066X.56.3.218

Harbaugh, W. T., Mayr, U., & Burghart, D. R. (2007). Neural responses to taxation and voluntary giving reveal motives for charitable donations. *Science*, *316*(5831), 1622–1625. https://doi.org/10.1126/science.1140738

Hayes, A. F. (2009). Beyond Baron and Kenny: Statistical mediation analysis in the new millennium. *Communication Monographs*, *76*(4), 408–420. https://doi.org/10.1080/03637750903310360

Kenny, D. A., Kashy, D. A., & Cook, W. L. (2006). *Dyadic data analysis*. Guilford Press.

Kerr, N. L., & Kaufman-Gilliland, C. M. (1994). Communication, commitment, and cooperation in social dilemma. *Journal of Personality and Social Psychology*, *66*(3), 513–529. https://doi.org/10.1037/0022-3514.66.3.513

Lemmer, G., & Gollwitzer, M. (2017). The “true” indirect effect won’t (always) stand up: When and why reverse mediation testing fails. *Journal of Experimental Social Psychology*, *69*, 144–149. https://doi.org/10.1016/j.jesp.2016.05.002

Raihani, N. J., & Smith, S. (2015). Competitive helping in online giving. *Current Biology*, *25*(9), 1183–1186. https://doi.org/10.1016/j.cub.2015.02.042

Reyniers, D., & Bhalla, R. (2013). Reluctant altruism and peer pressure in charitable giving. *Judgment and Decision Making*, *8*(1), 7–15.

Roberts, G. (1998). Competitive altruism: From reciprocity to the handicap principle. *Proceedings: Biological Sciences*, *265*(1394), 427–431.

Ryan, R. M., & Deci, E. L. (2000). Self-determination theory and the facilitation of intrinsic motivation, social development, and well-being. *American Psychologist*, *55*(1), 68–78. https://doi.org/10.1037/0003-066X.55.1.68

Scott, S. (1976). Practical reason and the concept of a human being. *The Journal of Philosophy*, *73*(15), 497–510. https://doi.org/10.2307/2025971

Sisco, M. R., & Weber, E. U. (2019). Examining charitable giving in real-world online donations. *Nature Communications*, *10*(1), 3968. https://doi.org/10.1038/s41467-019-11852-z

Spencer, S. J., Zanna, M. P., & Fong, G. T. (2005). Establishing a causal chain: Why experiments are often more effective than mediational analyses in examining psychological processes. *Journal of Personality and Social Psychology*, *89*(6), 845–851. https://doi.org/10.1037/0022-3514.89.6.845

Thoemmes, F. (2015). Reversing Arrows in Mediation Models Does Not Distinguish Plausible Models. *Basic and Applied Social Psychology*, *37*(4), 226–234. https://doi.org/10.1080/01973533.2015.1049351

Valeri, L., & VanderWeele, T. J. (2013). Mediation analysis allowing for exposure–mediator interactions and causal interpretation: Theoretical assumptions and implementation with SAS and SPSS macros. *Psychological Methods*, *18*(2), 137–150. https://doi.org/10.1037/a0031034

Weinstein, N., & Ryan, R. M. (2010). When helping helps: Autonomous motivation for prosocial behavior and its influence on well-being for the helper and recipient. *Journal of Personality and Social Psychology*, *98*(2), 222–244. https://doi.org/10.1037/a0016984

**Table S1. Summary of donation rates ($) for same-sex and cross-sex dyads.**

|  | Experiment 1 | | | |
| --- | --- | --- | --- | --- |
|  | Same-Sex Dyads | | Cross-Sex Dyads | |
| Condition | *n*_Dyads_ | *M* (*SD*) | *n*_Dyads_ | *M* (*SD*) |
| Collaborative Giving | 35 | 4.76 (.77) | 16 | 4.44 (1.26) |
| Independent Giving | 34 | 4.34 (1.04) | 16 | 4.59 (.90) |
| Total | 69 | 4.55 (.93) | 32 | 4.52 (1.08) |
|  | Experiment 2 | | | |
|  | Same-Sex Dyads | | Cross-Sex Dyads | |
| Condition | *n*_Dyads_ | *M* (*SD*) | *n*_Dyads_ | *M* (*SD*) |
| Collaborative Giving | 30 | 3.86 (1.43) | 21 | 4.04 (1.47) |
| Independent/Private Giving | 62 | 3.99 (1.61) | 42 | 3.75 (1.36) |
| Total | 92 | 3.94 (1.55) | 63 | 3.84 (1.39) |

**Table S2. Coding dimension reliabilities (ICCs), means and bivariate correlations (*r*s) between coding dimensions and each generosity ($) and intrinsic enjoyment for dyads assigned to the *Collaborative Giving* condition across Experiments 1 and 2.**

| **Coding**  **Reliability** | | **Experiment 1**  **(*n* = 37 dyads)** | | | **Experiment 2**  **(*n* = 45 dyads)** | | |
| --- | --- | --- | --- | --- | --- | --- | --- |
| **Coding Dimension** | ***ICC* (2,4)** | **Mean (SD)** | **Generosity**  $0 – $5 | **Intrinsic Enjoyment**  1 – 7 | **Mean (SD)** | **Generosity**  $0 – $5 | **Intrinsic Enjoyment**  1 – 7 |
| ***Advertisement Evaluation Phase*** | | | | | | | |
| **Discussion Time**  Minutes | - | 5.16  (4.07) | .09 | .28^†^ | 4.76  (2.42) | -.09 | -.01 |
| **Relevance**  % of time having relevant discussion  1 – 0%  5 – 100% | .70 | 4.86  (.20) | -.06 | .09 | 4.77  (.33) | .14 | .20 |
| **Depth**  1 – Not at all deep  5 – Very deep | .83 | 3.14  (.90) | .06 | **.49*^**^*** | 3.32  (.84) | .03 | **.34^*^** |
| **Dynamic**  1 – One-sided  5 – Lively back & forth | .85 | 3.45  (.95) | .09 | **.46*^**^*** | 3.45  (.86) | .12 | **.40*^**^*** |
| ***Joint Donation Decision Phase*** | | | | | | | |
| **Discussion Time**  Minutes | - | 1.46  (.68) | -.09 | .11 | 1.44  (1.03) | **-.48^***^** | -.23 |
| **Relevance**  % of time having relevant discussion  1 – 0%  5 – 100% | .69 | 4.80  (.43) | -.07 | .15 | 4.46  (.49) | .04 | -.22 |
| **Depth**  1 – Not at all deep  5 – Very deep | .87 | 1.97  (1.00) | .16 | .33^†^ | 2.24  (.92) | -.21 | .07 |
| **Dynamic**  1 – One-sided  5 – Lively back & forth | .82 | 2.85  (.98) | .12 | **.52*^**^*** | 3.00  (.93) | -.07 | .25 |

*Note.* ^†^ *p* < .10; *^*^p* < .05; *^**^p* < .01; *^***^p* < .001. Bolded correlations are statistically significant at *α* < .05.

Condition: Collaborative

(vs. independent)

Interpersonal Closeness

Intrinsic Enjoyment

Indirect Effect = .39 [.21, .61]

*b* =.59^***^

*b* =.67^**^

*b* = .55^**^

*b*` = .16

Condition: Collaborative

(vs. independent & private)

)

Interpersonal Closeness

Intrinsic Enjoyment

Indirect Effect = .53 [.33, .75]

*b* =.72^***^

*b* =.74^***^

*b* = .36^*^

*b*` = -.17

**Experiment 1**

**Experiment 2**

Figure S1. The indirect effect of collaborative giving (vs. individual giving) on intrinsic enjoyment through interpersonal cloeseness in Experiments 1 and 2.

*Note.* All *b*’s represent unstandardized regression coefficients. Path a was estimated using Multilevel Modeling (MLM) with Maximum Likelihood estimation. The MLM models estimating path b did not converge and there was no evidence of non-independence in intrinsic enjoyment in either experiment, thus path b was estimated using OLS regression The indirect effect was obtained using *RMediation*. The range in brackets represents the 95% confidence interval of the indirect effect. *^*^p* < .05*; ^**^p* < .01; otherwise, *p* > .05.

1. We additionally included the response option for participants to identify as “non-binary” or to self-report their gender identity across studies. Only one person in Experiment 2 identified as genderqueer. Thus, this dyad was excluded from the analyses because we do not have the statistical power to make strong conclusions about how a non-binary gender identity might interact with condition to predict generosity. [↑](#footnote-ref-1)
2. We initially had a sample of 38 dyads with video footage, but one dyad in Experiment 1 spoke in a language other than English and could not be coded by our English-speaking coders; this dyad was removed from our analyses, leaving a total of 37 dyads. One dyad in Experiment 1 completed the advertisement evaluation phase but not the joint-donation decision phase of the task; the available codes from this dyad were included in our analyses. [↑](#footnote-ref-2)
3. One item of the relatedness subscale, which had reduced overall scale reliability in Experiment 2 was removed. Including this item did not substantially change reliability in either study or the conclusions of the analyses. [↑](#footnote-ref-3)
4. As above, to maximize statistical power, we collapsed across the *independent* and *private giving* conditions in Experiment 2. [↑](#footnote-ref-4)
